# The Role of Platelet Factor 4 in Local and Remote Tissue Damage in a Mouse Model of Mesenteric Ischemia/Reperfusion Injury

**DOI:** 10.1371/journal.pone.0039934

**Published:** 2012-07-06

**Authors:** Peter H. Lapchak, Antonis Ioannou, Poonam Rani, Linda A. Lieberman, Kazuhisa Yoshiya, Lakshmi Kannan, Jurandir J. Dalle Lucca, M. Anna Kowalska, George C. Tsokos

**Affiliations:** 1 Rheumatology Division, Beth Israel Deaconess Medical Center and Harvard Medical School, Boston, Massachusetts, United States of America; 2 Department of Pediatrics, University of Pennsylvania School of Medicine, Children’s Hospital of Philadelphia, Philadelphia, Pennsylvania, United States of America; 3 The United States Army Institute of Surgical Research, San Antonio, Texas, United States of America; French National Centre for Scientific Research, France

## Abstract

The robust inflammatory response that occurs during ischemia reperfusion (IR) injury recruits factors from both the innate and adaptive immune systems. However the contribution of platelets and their products such as Platelet Factor 4 (PF4; CXCL4), during the pathogenesis of IR injury has not been thoroughly investigated. We show that a deficiency in PF4 protects mice from local and remote tissue damage after 30 minutes of mesenteric ischemia and 3 hours of reperfusion in *PF4-/-* mice compared to control B6 mice. This protection was independent from Ig or complement deposition in the tissues. However, neutrophil and monocyte infiltration were decreased in the lungs of *PF4-/-* mice compared with B6 control mice. Platelet-depleted B6 mice transfused with platelets from PF4-/- mice displayed reduced tissue damage compared with controls. In contrast, transfusion of B6 platelets into platelet depleted *PF4-/-* mice reconstituted damage in both intestine and lung tissues. We also show that PF4 may modulate the release of IgA. Interestingly, we show that PF4 expression on intestinal epithelial cells is increased after IR at both the mRNA and protein levels. In conclusion, these findings demonstrate that may PF4 represent an important mediator of local and remote tissue damage.

## Introduction

Ischemia reperfusion (IR) injury is defined as tissue damage occurring after a transient loss of blood supply and subsequent return [1]. During this process an extensive activation of the inflammatory response first locally and then to almost all remote organs leading to tissue damage [2]. Complement activation, natural Ig, neutrophils, T cells and other immune mediators have been shown to play a significant role in this process [3]–[6].

It has been well documented that natural IgM antibodies, self-reactive IgM antibodies and local complement activation are necessary to induce tissue damage after IR injury [3], [7]–[9]. In contrast, there is little is known on the role of IgA in tissue damage after IR injury. It has been reported, however, that although IgA is a poor activator of complement due to its inability to bind C1q, it can initiate complement activation via the alternate pathway [10]. Our group has recently showed that IgA deposition is increased locally in the intestine and also remotely in the lung after mesenteric IR injury in C57BL/6J mice [11]. Therefore, natural IgM and mucosal IgA may initiate tissue injury using different but overlapping complement activation pathways.

Platelets or platelet-derived factors have been shown to modulate the inflammatory response in many clinical entities including chronic and acute inflammatory responses in rheumatoid arthritis [12] systemic lupus erythematosus [13], inflammatory bowel disease [14], vascular inflammation in graft rejection [15] and more recently in ischemia reperfusion injury [16]. Platelets are activated through their interaction with integrins [17]. Upon activation, platelets release many different molecules, including the chemokine CXCL4 or Platelet factor-4 (PF4), a 70-amino acid protein that exists as a 150 KDa tetramer. PF4 comprises 2–3% of the total platelet proteins and 25% of the total á-granule proteins and is released during platelet activation [18]–[20]. PF4 inhibits local antithrombin III activity by binding with high affinity to heparin-like molecules thus promoting coagulation. PF4 is involved in many other biological processes including promoting survival of hematopoietic stem cells [21] and inhibiting proliferation of endothelial cells and fibroblasts [22]. More recently, it has been shown that PF4 forms stable heterodimers with CCL5 (RANTES) resulting in a synergistically amplified ability to recruit monocytes [23]. Importantly, it has been shown that during inflammation PF4 is able to promote the adherence of neutrophils onto endothelial cells, promote neutrophil exocytocis [24], and supports the generation of reactive oxygen species and other pro-inflammatory cytokines from monocytes [25], [26], [27] to maintain the immune response.

The generation of both PF4 deficient mice (PF4-/-) on a B6 background and mice overexpressing human PF4 has shed light to its central role in thrombus formation and atherosclerosis [19]. Importantly, PF4 deficient mice do not have bleeding diathesis and their white and red blood cell counts and hematocrit levels are within normal range. Moreover, introduction of a PF4 null locus, into the ApoE-/- mouse, a well known model of atherosclerosis reduced atherosclerotic lesion formation compared with control mice, thus demonstrating the important role of PF4 in the pathogenesis of atherosclerosis [28].

Here we report here that a deficiency in PF4 can mitigate tissue injury in the intestine and lung following mesenteric IR injury.

## Materials and Methods

### Ethics Statement

All experiments were performed in accordance with the guidelines and approval of the Institutional Animal Care and Use Committee of the Beth Israel Deaconess Medical Center.

### Mice

PF4-/- mice on a B6 background were generated as previously reported [19], (Children’s Hospital of Philadelphia) and backcrossed onto a C57BL/6J background (N>20). C57BL/6J were purchased from The Jackson Laboratory (Bar Harbor, ME) and housed in the animal research facility at the Beth Israel Deaconess Medical Center (BIDMC) and allowed to acclimate for 7 days prior to their use in experiments. Eight to twelve week old male mice were used for all the experiments. All experiments were performed with age-matched PF4-/- mice and C57BL/6J.

### Ischemia Reperfusion Injury Protocol

Mice were randomly assigned to either sham or IR groups. was used for anesthesia. Anesthesia was induced by intraperitoneal (IP) administration of pentobarbital (72 mg/kg; Nembutal, Lundbeck Inc., Deerfield, IL) and maintained thereafter with a 36 mg/kg dose. Mice were subjected to IR injury as previously described [11]. Briefly, a midline laparotomy was performed; the superior mesenteric artery was identified, isolated, and clamped using a small non-traumatic micro vascular clip. After 30 minutes the clip was removed and the intestines were allowed to reperfuse for up to 3 hours. The sham group was subjected to the same surgical intervention without artery occlusion. The laparotomy incision was sutured, the mice resuscitated with 1.0 mL pre-warmed sterile PBS subcutaneously and body temperature was maintained at 37°C for the duration of the experimental procedure. After 3 hours, mice were euthanized by carbon dioxide asphyxiation and whole blood and tissues were harvested. Intestine was removed, flushed with ice-cold PBS and 10% phosphate-buffered formalin. The specimens were fixed in 10% phosphate-buffered formalin at 4°C overnight. Intact lungs and bronchial tree was isolated, expanded with 200–300 µL of 10% phosphate-buffered formalin, removed. and fixed overnight at 4°C in 10% phosphate-buffered formalin. Whole blood was obtained from sham control mice and mice that underwent mesenteric ischemia reperfusion by cardiac puncture in to tubes containing 168 mM dipotassium EDTA. Plasma was prepared and stored at −80°C for additional studies.

### Platelet Depletion

Mice received a single IP injection of an affinity purified endotoxin-free rabbit anti-mouse polyclonal antibody prepared with commercially available rabbit anti-mouse platelet anti-sera (Inter-Cell Technologies, Jupiter, FL) as described previously [29]. For experiments, C57BL/6J mice were depleted of platelets two days prior to and ischemia reperfusion experiments and were randomly assigned to either sham or mesenteric IR groups.

### Platelet Isolation and Transfusion

Whole blood was collected into syringes containing acid citrate dextrose by cardiac puncture and transferred to polypropylene tubes. After centrifugation at 100 X g at room temperature, the upper phase containing platelet rich plasma was removed and the platelets pelleted and resuspended in Tyrodes’ buffer for transfusion as described previously [30]. Platelet numbers were determined using Hemavet 850 (Drew Scientific, Farmington, CT). Platelets were transfused into anesthetized platelet-depleted recipient mice ten minutes prior to the start of experiments.

### ELISA

Plasma samples from sham control mice and mice that underwent mesenteric ischemia reperfusion were prepared and used in the mouse CXCL4/PF4 ELISA (cat. # DY595, R&D Systems, Inc., Minneapolis, MN) according to the manufacturer’s specifications.

### Ig Isotyping

Plasma samples from sham control mice and mice that underwent mesenteric ischemia reperfusion were prepared and used in the multiplex mouse Ig isotyping kit (cat. # MGAM-300, Millipore, Inc., Minneapolis, MN) according to the manufacturer’s specifications. The sensitivity/accuarcy of the kit is as follows: IgG1 0.7 ng/mL/>IgG1 96%, IgG2a 0.4 ng/mL/>IgG2a 98%, IgG2b 0.6 ng/mL/>IgG2b 85%, IgG3/>IgG3 76%, 0.8 ng/mL, IgA 1.1 ng/mL/>IgA 100%, and IgM 0.5 ng/mL/>IgM 98%.

### Real-time Quantitative Reverse Transcriptase-polymerase Chain Reaction (RT-PCR)

Villi from the small intestine were isolated from the submucosa by gentle scraping with a sterile microscope slide and lung tissues were transferred to tubes containing TRIZOL reagent immediately after harvest and stored at −80°C until RNA purification. Total RNA was isolated from small intestine and lung using TRIZOL reagent (Invitrogen, Carlsbad, CA). cDNA was synthesized using the high capacity cDNA reverse transcription kit (Applied Biosystems, Carlsbad, CA) according to manufacturer’s protocols. Real time RT-PCR was performed with LightCycler 480 System (Roche, South San Francisco, CA) using TaqMan gene expression Master Mix and predesigned TaqMan probes for mouse PF4 and GAPDH as recommended by Applied Biosystems. The averaged cycle threshold values of each reaction derived from the target gene, determined with LightCycler 480 System software (Roche, South San Francisco, CA), and were normalized to GAPDH levels. Cycle threshold values were used to calculate relative mRNA expression by the ÄÄCt relative quantification method.

### Histology and Tissue Injury Scoring

Formalin-fixed intestine and lung tissues were processed and embedded in paraffin for histological analysis. Intestine and lung sections of 5–8 µm thickness were stained with hematoxylin and eosin and subjected to histological scoring to evaluate tissue damage. All histological analysis was performed in a blinded manner.

For each intestinal section, 100 villi were graded using a 6-tiered scale as described previously [31]. Briefly, a normal appearing villus was assigned a score of 0 while villi demonstrating tip distortion were scored as 1. Villi without goblet cells and with Guggenheims’ spaces were scored as 2 and villi containing patchy disruption of the epithelial cells were scored as 3. Villi demonstrating exposed, intact lamina propria and sloughing of epithelial cell were scored as 4. Villi demonstrating exuding lamina propria were assigned a score of 5, and lastly, villi with hemorrhage or denudation were scored as 6. Scoring of lungs for alveolar and peri-luminal injury was calculated based on Cooke’s method [32]. Ten to twenty fields at high power field magnification (400×) were viewed for each lung section and scored for alveolar infiltration on a 3-tiered scale. The following calculation for alveolar scores was performed as follows: a score of 0 was given when no infiltrate was present; a score of 1 was given when the infiltrate could be visualized easily only at 400×; when infiltrates were readily visible, a score of 2 was assigned; and the score for consolidation was 3. Similarly, each section was scored for peri-luminal damage (airway or blood vessel) at 100×. The calculation for peri-luminal scores was as follows: when there was no infiltrate a score of 0 was assigned; when the infiltrate was between 1 and 3 cell layers thick, the score was 1; for infiltrates ranging from 4 to 10 cells layers thick; a score of 2 was assigned; and infiltrates >10 cell layers thick were scored as 3. Based on the overall involvement of the section, a severity score was calculated: the severity score for 0–25% involvement was 1; a severity score of 2 was assigned for 25–50% involvement; and the severity score for >50% involvement was 3. For calculation of the total lung injury score, the means of alveolar and peri-luminal scores for each section for summed up and multiplied by the severity score, which gave a final score ranging from 0 to 18.

### Immunohistochemistry

For Immunohistochemistry, formalin-fixed paraffin sections of intestine and lung were subjected to rehydration and antigen retrieval as described by the manufacturer (BD Biosciences, Billerica, MA). After blocking with PBS+10% FCS for an hour, the sections were incubated overnight with primary antibodies. The following day, sections were washed and incubated with secondary antibodies for 1 h. Stained sections were developed with NovaRed (Vector Laboratories, Burlingame, CA) and counterstained with hematoxylin (Vector Laboratories, Burlingame, CA). Appropriate isotype controls were used. For immunohistochemical studies, the following reagents were used: affinity-purified rabbit, rat and goat polyclonal antibody, rabbit anti-mouse C3 (B-9,Santa Cruz Biotech, Santa Cruz, CA), goat anti-mouse IgA (Invitrogen, Carlsbad, CA), goat anti-mouse IgG (Abcam, Cambridge, MA ), goat anti-human PF4, peroxidase-conjugated affinity-purified secondary antibodies to rabbit rat and goat immunoglobulin (Jackson ImmunoResearch, West Grove, PA)**,** rat monoclonal anti-Neutrophil (ab2557, Abcam, Cambridge, MA) and rat monoclonal anti-Monocyte (ab15636, Abcam, Cambridge, MA).

### Neutrophil and Monocyte Infiltration Scoring

Sham control and experimental lung tissues stained for monocytes or neutrophils as described in the Immunohistochemistry section above were scored for neutrophil and monocyte infiltration. Positive staining cells were counted in 20 fields at 100× or 200× for total lung tissue and the average was calculated and plotted.

### Image Development

Prepared slides were viewed on a Nikon Eclipse 80 i microscope, and images were taken using the Nikon DS-FiI digital camera and saved as tiff files. Images were adjusted globally using the adjustment feature in the RGB channel using Adobe Photoshop CS2 (Adobe Systems, San Jose, CA).

### Statistical Analysis

Data are presented as mean ± SD. All data were subjected to statistical analysis using GraphPad Prism 4.0 for Windows software program (GraphPad Software, San Diego, CA). A p≤0.05 was considered significant using the student t test to compare sham and IR groups.

## Results

### PF4 Deposition is Dramatically Increased in the Lung and Intestine of B6 Mice after Mesenteric IR Injury

**Figure 1:**
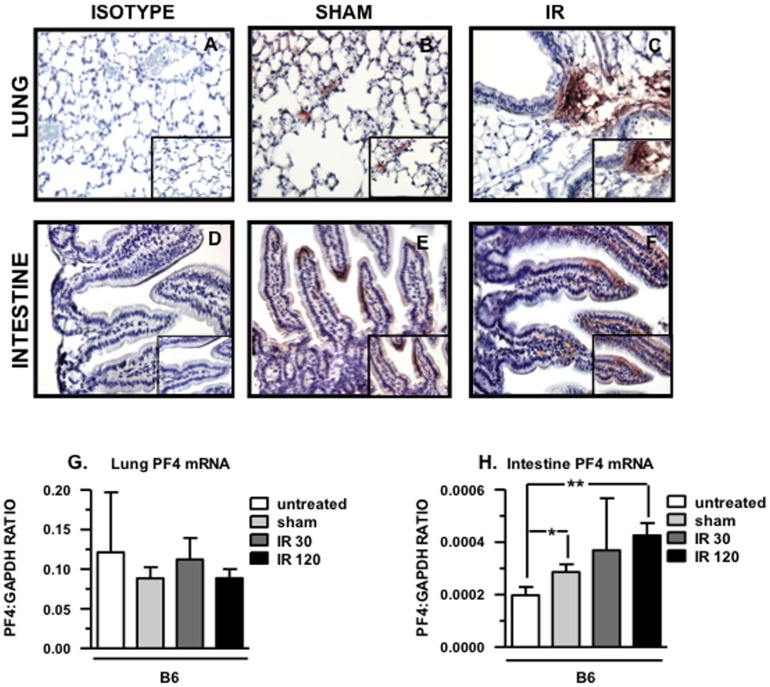
PF4 deposition increased dramatically in the intestine and lung of B6 mice after mesenteric IR injury. Tissue sections of lung (A-C) and intestine (D-F) from B6 after 30 minutes of mesenteric ischemia and 3 hrs of reperfusion were stained for PF4 (red) and counterstained with hematoxylin (blue). Images are representative of 3–4 mice per group. (G) PF4 mRNA levels in lung. (H) PF4 mRNA levels in intestinal epithelial cells. All images shown are 200× and 400× magnification. *p≤0.05, **p≤0.01, for IR compared to sham controls.

Because PF4 mediates adherence and pro-inflammatory functions of neutrophils and monocytes and both cell types are invariably present in damaged tissue after mesenteric IR injury [2] we stained intestinal and lung tissue sections B6 mice that undergone mesenteric IR injury with an anti-PF4 antibody. As shown in figure 1A–F, abundant amount of PF4 is detected in the lung vasculature and in the intestinal villi of B6 mice that underwent mesenteric IR injury compared to sham-operated mice. Furthermore, we found that PF4 deposition in the intestine was limited to the intestinal epithelial cells of the villi. To exclude the possibility that PF4 protein in the intestine was platelet-associated and to verify that it was produced by IEC, we stained for PF4 in intestine and lung tissue sections obtained from platelet depleted mice that were either sham-treated or had undergone mesenteric IR injury (Figure S1B, C). PF4 deposition in the lung was reduced compared to sham and IR platelet-intact mice (Figure 1 B, C). In the intestine, PF4 protein was detected in the villi, was predominantly found at the tips (Figure S1E) and was both more widespread and increased after mesenteric IR injury (Figure S1F). Together, these findings suggest that intestinal epithelial cells may be a novel source of PF4 and may play a role in tissue damage following mesenteric IR injury.

Since we identified PF4 deposition in the villi of the small intestine in sham-treated mice, and this deposition dramatically increased after mesenteric IR injury, mRNA levels were determined from isolated villi and from lung by real time PCR. As shown in figure 1H, mRNA levels obtained from isolated villi increased in sham controls when compared to naïve (untreated) mice (p≤0.05) and this expression further increased after 30 minutes of ischemia and 120 minutes of reperfusion (p≤0.01). In contrast, there are no differences in mRNA levels in the lungs from naïve mice, sham-treated mice or mice that underwent the experimental procedure (Figure 1G).

### Intestine and Lung Tissue Damage is Reduced in PF4-/- Mice after Mesenteric IR Injury

**Figure 2:**
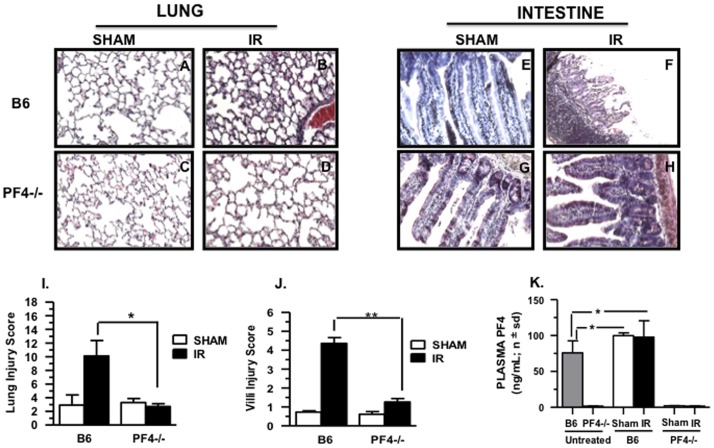
Intestinal and lung injury is reduced after mesenteric ischemia/reperfusion in PF4^-/-^ mice: Hematoxylin and eosin stained sections of mouse small intestine and lung after 30 minutes of ischemia and 3 hours reperfusion. A total of 5–8 mice were used for each control and experimental groups in two experiments. (A-D) Images of lung from sham and IR (E-H). Images of intestinal villi from sham and IR. All images shown are 200× magnification. (I,J) Injury score (mean ± SD) in lung and intestine.(K) PF4 plasma levels in B6 and PF4-/- mice before and after mesenteric IR injury. *p≤0.05, **p≤0.01, and ***p≤0.001 for IR compared to sham controls.

To evaluate the role of PF4 in tissue damage, PF4 deficient mice were subjected to mesenteric IR injury. As can be seen in Figure 2A–H, lung and intestinal damage in *PF4-/-* was reduced compared to wild type B6 control mice. Moreover, there was a significant reduction in intestinal and lung injury scores compared to B6 mice (Figure 2I, J). We verified that PF4 protein is absent in PF4−/− mice under all experimental conditions as shown in Figure 2K. In contrast, plasma PF4 levels were found in naïve C57BL/6J mice and were significantly increased in both sham-treated mice and mice that underwent mesenteric IR injury (Figure 2K). This increase in plasma PF4 levels in both sham and IR B6 mice may be attributed to platelet activation and release of á-granules contents following mild trauma (midline laparotomy) or major trauma (midline laparotomy and IR).

### Neutrophil (PMN) and Monocyte (MO) Infiltration is Reduced in the Lung of PF4-/- Mice after Mesenteric IR Injury

**Figure 3:**
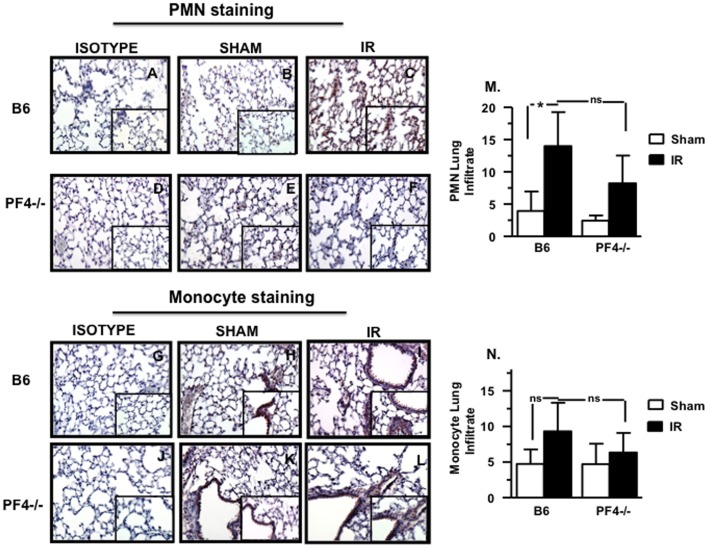
Neutrophil (PMN) and monocyte infiltration is reduced in the lung of PF4-/- mice after mesenteric IR injury. Tissue sections of lung of B6 and PF4-/- mice after 30 minutes of mesenteric ischemia and 3 hrs of reperfusion and were stained for neutrophils (A-F, red) and monocytes (G-L, red) and counterstained with hematoxylin (blue). A total of 5–8 mice were used for each control and experimental groups in two experiments. (M) Neutrophil infiltration score, (N) Monocyte infiltration score. ns: not significant *p≤0.05 for IR compared to sham controls. Red: Positive Staining.

PF4 has been reported to modulate the function of PMN and monocytes [23]–[27]. To further evaluate the role of PF4 in IR injury, intestine and lung sections were stained for PMN and monocytes and these cells counted to determine increased infiltration. We found that both PMN (Figure 3A–F) and MO infiltration (Figure 3G–L) into lungs were reduced in *PF4-/-* mice when compared to B6 mice. Cumulative data shown in Figure 3 M, N suggest a trend lower but which did not reach statistical significance. However, when we compared B6 mice that underwent mesenteric IR compared to sham control mice, PMN infiltration was significantly greater. In contrast, no differences were observed in PMN and MO infiltration in the intestinal tissue from both *PF4-/-* and B6 mice (Figure S2). This finding suggests that PF4 may be partially involved in neutrophil and monocyte recruitment into the lung leading to remote tissue damage after mesenteric IR injury.

### Complement and Immunoglobulin (Ig) Deposition in PF4-/- Mice

**Figure 4:**
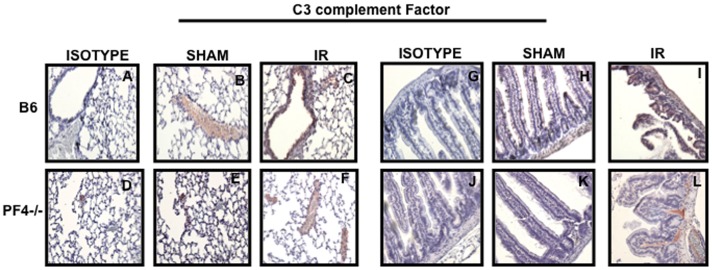
Tissue damage in PF4-/- mice is not associated with complement deposition. Tissue sections of lung (A-F) and intestine (G-L) from B6 and PF4-/- mice after 30 minutes of mesenteric ischemia and 3 hrs of reperfusion and were stained C3 complement factor (red) and counterstained with hematoxylin (blue). Images are representative of 3–4 mice per group.

**Figure 5:**
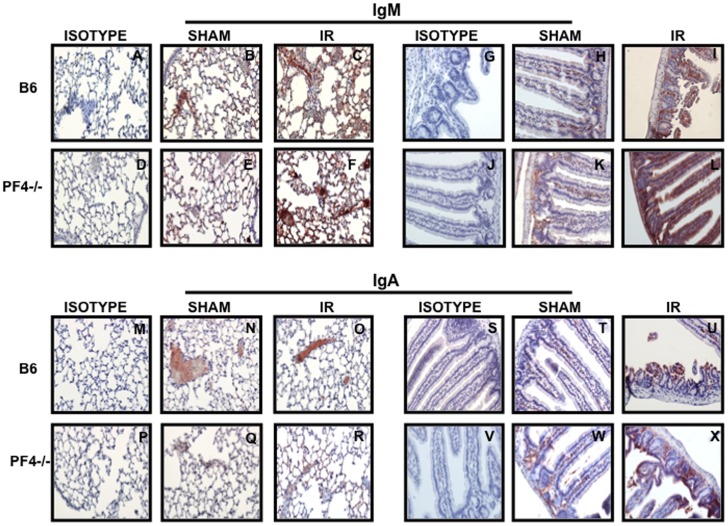
Tissue damage in PF4-/- mice is not associated with immunoglobulin (Ig) deposition. Tissue sections of lung and intestine from B6 and PF4-/- mice after 30 minutes of mesenteric ischemia and 3 hrs of reperfusion were stained for IgM (A-L, red) and IgA (M-X, red) and counterstained with hematoxylin (blue). Images are representative of 3–4 mice per group. Red: Positive Staining.

It has been reported that complement and immunoglobulin (Ig) deposition is associated with tissue damage after mesenteric IR injury [3]. Therefore, we next examined the presence of complement and Ig in intestinal and lung tissue sections from *PF4-/-* mice subjected to mesenteric IR injury. Surprisingly, we did not find differences in complement (Figure 4A–L), IgM (Figure 5A–L) and IgA (Figure 5M–X) deposition between PF4-/- and B6 control mice. We conclude that in the PF4 deficient mice complement and Ig deposition alone is not sufficient to induce tissue damage after mesenteric IR injury and that additional components are required.

**Figure 6:**
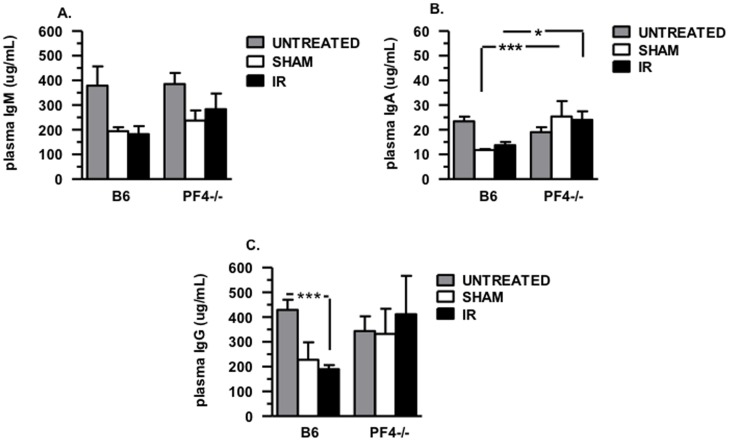
Immunoglobulin (Ig) plasma levels in B6 and PF4-/- mice before and after mesenteric IR injury. Plasma samples obtained from B6 and PF4-/- mice before and after mesenteric IR injury were subjected to multiplex analysis. (A) IgM plasma levels. (B) IgA plasma levels, (C) IgG plasma levels. ns: not significant *p≤0.05, **p≤0.01, and ***p≤0.001 for IR compared to sham controls. A total of 5–8 mice were used for each control and experimental groups in two experiments.

Next we addressed whether a deficiency in PF4 leads to altered immunoglobulin production. Plasma samples from untreated, sham and IR B6 and PF4-/- mice were subjected to multiplex analysis. There were no significant differences in plasma levels of total IgM (Figure 6A), IgA (Figure 6B) and IgG (Figure 6C) in untreated naive C57BL/6J and PF4-/- mice. We next determined plasma Ig (IgG, IgM and IgA) levels in sham control mice and in mice that underwent mesenteric ischemia reperfusion. We found no significant differences in total plasma IgM (Figure 6A) and IgG (Figure 6C) in the sham control or mesenteric IR C57BL/6J and PF4-/- mice. Surprisingly, we did find that plasma IgA levels in PF4-/- sham control mice and mice that underwent mesenteric IR injury were significantly increased (p≤0.01 and p≤0.05, respectively) compared to their C57BL/6J experimental counterparts (Figure 6A). These data suggest that PF4 may regulate IgA release from IgA producing B lymphocytes in mucosal tissues.

### Transfusion of PF4-/- Platelets into Platelet Depleted B6 Mice Fails to Restore Local and Remote Tissue Damage after Mesenteric IR Injury

**Figure 7:**
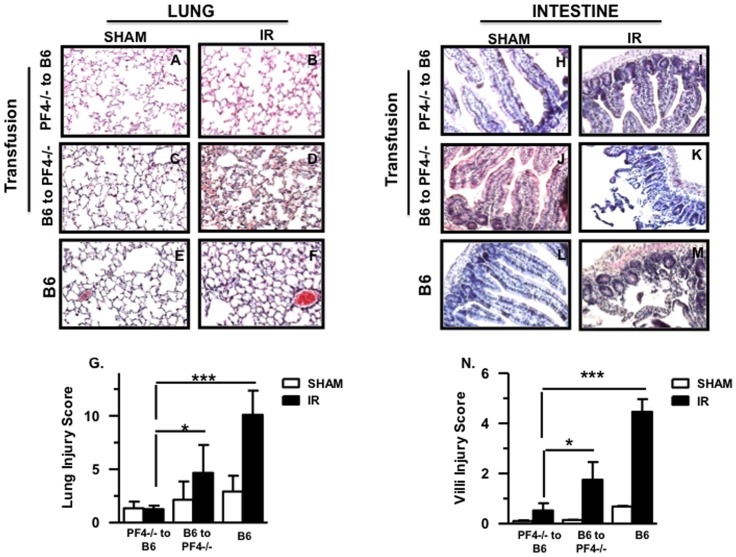
Transfusion of PF4-/- platelets into platelet depleted B6 mice fails to restore local and remote tissue damage after mesenteric IR injury. Hematoxylin and eosin stained lung and small intestine sections of PF4-/- mice transfused with B6 platelets and vise versa after 30 minutes of ischemia and 3 hours reperfusion. A total of 5–8 mice were used for each control and experimental groups in a total of two experiments. (A-F) Images of lung from sham and IR. (H-M) Images of small intestine from sham and IR. All images shown are 200× magnification. (G,N) Injury score (mean ± SD) in lung and small intestine respectively. *p≤0.05 and ***p≤0.001 for IR compared to sham controls.

It is well known that PF4 is predominantly synthesized in megakaryocytes and stored in platelet á-granules [17]. Accordingly we asked whether platelet-derived PF4 accounts for the tissue damage after mesenteric IR injury. We transfused purified platelets from *PF4-/-* mice into platelet-depleted B6 mice and performed mesenteric IR injury. Normal B6 and platelet-depleted *PF4-/-* mice transfused with B6 platelets were used as controls. Platelet-depleted B6 mice transfused with *PF4-/-* platelets displayed reduced lung (Figure 7A–B) and intestinal (Figure 7H–I) tissue damage. In contrast, transfusion of B6 platelets into platelet depleted *PF4-/-* mice reconstituted a statistically significant amount of damage in both lung (Figure 7C–F) and intestine (Figure 7J–M). Cumulative data are shown in Figures 7G and Figure 7N for lung and intestine respectively. In conclusion, these experiments confirm that platelet-derived PF4 plays a definitive role in the expression of remote tissue damage after mesenteric IR injury.

## Discussion

In this study we introduce a novel role for PF4 as a mediator of remote lung injury after mesenteric IR injury. We first demonstrate that intestinal epithelial cells (IEC) express low basal levels of PF4 mRNA (and protein) in sham-treated mice and this expression is dramatically increased after 120 min of reperfusion. In contrast there was no increase in PF4 mRNA in lung under any experimental condition. To our knowledge, there are no reports in the literature describing PF4 expression in the gut (IEC) as it was initially thought to be platelet specific, although it is now documented that other immune cells also produce it, albeit at much lower levels [33]. Furthermore, this is the first demonstration that ileal IEC are capable of producing PF4. We postulate that PF4 mRNA expression and protein in the gut may be important in gut homeostasis. PF4 combines with other chemokines such as RANTES (CCL5), [23] and forms heterodimers. These heterodimers lead to enhanced neutrophil and monocyte chemoattraction. Hence, low levels of PF4 in combination with other chemokines may constantly recruit leukocytes to the villi to maintain barrier function. The increase in IEC PF4 protein and mRNA after mesenteric IR injury may provide an alarm signal and as mentioned, form heterodimers with other chemokines, recruits neutrophils and monocytes to protect the host from bacterial invasion due to the loss of barrier integrity.

Since PF4 in combination with RANTES (CCL5) has been shown to recruit PMN and monocytes [23], we determined whether there were differences in PMN and monocyte recruitment in lung and intestine after mesenteric IR injury in B6 and PF4-/- mice. We found that PMN numbers were significantly increased in lung after IR injury in B6 mice. In contrast, there were no significant differences in the numbers infiltrating PMN in PF4-/- mice. Recently we published a report demonstrating a role for platelets in remote lung injury following mesenteric IR injury [16]. Platelet depleted mice were devoid of any remote tissue damage, however, villi injury although reduced was still significantly greater when compared to sham controls. Reconstitution of platelet-depleted B6 mice re-established lung damage. Because PF4 is a major á-granule product of platelets [17], locally high levels of PF4 in the lung vasculature subsequent to platelet stasis after mesenteric IR injury may result in a chemokine gradient that is selective for recruiting PMN and monocytes to this site. Once attracted to these sites, PF4 also induces PMN and MO adherence to endothelial cells on the vascular wall and their eventual diapedesis into the lung proper. It has been shown by Woller et al that PF4 stimulates human monocytes to release reactive oxygen species (ROS). ROS has been shown to initiate endothelial cell apoptosis [27]. Therefore, PF4-activated monocytes in the intestine and lung of mice after IR could initiate endothelial cell apoptosis. In contrast, we previously found minimal numbers of platelets in villi after injury at any time point tested [16]. Locally produced PF4 by IEC at or near the site of injury may be sufficient to recruit IgA producing B cells to the site. Hence, our findings suggest a direct role for PF4 in recruiting PMN to sites of remote injury. PF4 by itself may exhibit limited chemotactic function, however, when combined with RANTES (CCL5) to form heterodimers, is much more potent. This may also be true for RANTES [34]. Moreover, activated platelets also release RANTES [34]–[38] and it has been reported that RANTES is only minimally effective in recruiting PMN and monocytes [34]. Thus our findings demonstrate a positive interaction between PF4 and RANTES and further positions PF4 as a critical mediator of remote tissue damage following mesenteric IR injury. Additional studies to investigate the role of RANTES alone or RANTES:PF4 are particularly warranted. The inability of platelets transfused from PF4-/- mice into platelet depleted B6 mice further supports our claim for a role of PF4 in local and remote tissue damage. As discussed above RANTES alone is only minimally chemotactic for neutrophils and monocytes, however, when combined with PF4 in heterodimers is a potent chemoattractant.

We next show that PF4-/- mice are protected from intestinal and lung tissue damage after mesenteric IR injury compared to B6 mouse controls. A number of studies by other and us have demonstrated a role for natural Ig in the injury process after mesenteric IR [3], [11]. We also sought to characterize in part, the innate response in PF4-/- mice. Hence, we evaluated plasma Ig levels, tissue bound Ig and complement deposition after mesenteric IR injury. We demonstrate that PF4 deficiency does not influence total Ig levels circulating in plasma or complement deposition. We next looked at the long established IgM and our recently reported IgA in mesenteric IR injury [11]. Surprisingly, plasma IgA levels were significantly increased in PF4-/- mice that were sham-treated and mice that underwent mesenteric IR injury although levels were similar in naïve PF4-/- mice when compared to B6 mice. These findings are novel since there has been no demonstrable role for PF4 in modulating IgA production. Since IgA exists as two separate immunoglobulin types, secreted and membrane-associated [39], we postulate that in the absence of PF4, there is an upregulation of secreted IgA which might be protective. Our postulation is based on the fact that there is reduced villi damage after mesenteric IR injury in PF4-/- mice, yet IgA deposition is similar on villi compared to that observed with B6 mice. Additional work will be necessary to address the role of PF4 on IgA production and release. No reports attribute a role for PF4 on B cell function, activation or Ig release. However, it has been reported that there is differential expression of chemokine receptors on human IgA+ and IgG+ B lymphocytes [40] with both B cell subsets expressing CXCR3 and CCR5. Hence there may be differential effects of RANTES:PF4 via CXCR3 and RANTES via CCR5 receptors. However, this study was limited since IgG and IgA production in response to these agonists were not determined. It is plausible that PF4 (in combination with RANTES to form heterodimers) may act as a chemokine to recruit IgA+ B lymphocytes to sites of inflammation while RANTES alone may act as an inducer of IgA release. Further studies are needed to demonstrate a link between these chemokines and Ig release.

We demonstrate that PF4 may directly or indirectly participate in remote tissue damage. We also describe a previously unknown source of PF4, the intestinal epithelial cell, and demonstrate that PF4 may directly or/indirectly alters IgA production. We next wanted to demonstrate that platelet-derived PF4 is involved in remote tissue damage. This was accomplished by transfusing platelets from PF4-/- mice to platelet depleted B6 mice. This led to significantly less lung damage and reduced intestine damage compared to the B6 control mice, an observation we previously made with mice depleted of platelets prior to mesenteric IR injury [16]. In both cases, the levels of tissue damage were similar. More importantly, transfusion of normal B6 platelets into platelet-depleted PF4-/- mice re-established tissue damage in both the lung and intestine. Hence PF4 either directly or indirectly through heterodimer formation with RANTES is a necessary participant in remote tissue damage. Release of PF4 from activated B6 platelets in PF4-/- mice was sufficient to re-establish remote lung tissue damage.

Increasing data support a central role for platelets and their products in the pathogenesis of many inflammatory diseases [41]–[44]. For example, in patients with systemic lupus erythematosus, it has been demonstrated that platelets are activated by circulating immune complexes and can modulate the recruitment of monocytes contributing to the severity of the disease [13]. Similarly in rheumatoid arthritis, P-selectin expressed on activated platelets regulates neutrophil recruitment at the site of inflamed tissue, thus contributing to the tissue damage [12]. Platelet-derived PF4 plays major role in endothelial proliferation and endothelial cell apoptosis, angiogenesis [45], tumor development [46] and atherosclerosis [19], [27], [28], [47], [48]. PF4 also has been found to influence granulocyte activation by increasing neutrophil adherence to endothelial cells, promoting the release of myeloperoxidase and lysozyme from these cells [49], protecting monocytes from spontaneous apoptosis [50] and affecting macrophage activation and differentiation [19], [51]. The regulation of these processes grants PF4 an important role during the inflammatory response. However the significance of this molecule and its biological functions have not been evaluated during mesenteric IR injury until know.

From this study, we propose a novel role for PF4 in the expression of tissue damage during mesenteric IR injury. Based on our findings we can suggest that PF4 is a potential therapeutic target against tissue damage during mesenteric IR injury.

## Supporting Information

Figure S1 **PF4 deposition in the lung and intestine of platelet depleted B6 mice after mesenteric IR injury.** Tissue sections of lung (A-C) and intestine (D-F) from platelet-depleted B6 after 30 minutes of mesenteric ischemia and 3 hrs of reperfusion were stained for PF4 (red) and counterstained with hematoxylin (blue). Images are representative of 3–4 mice per group. All images shown are 200× and 400× magnification.
(TIF)

Figure S2 **Neutrophil (PMN) and monocyte infiltration in intestine of PF4-/- mice and B6 after mesenteric IR injury.** Tissue sections of intestine of B6 and PF4-/- mice after 30 minutes of mesenteric ischemia and 3 hrs of reperfusion and were stained for neutrophils (A-F, red) and monocytes (G-L, red) and counterstained with hematoxylin (blue). A total of 5–8 mice were used for each control and experimental groups in two experiments. All images shown are 200× and 400× magnification.
(TIF)
