# Supplementary figures and images for: The Role of Platelet Factor 4 in Local and Remote Tissue Damage in a Mouse Model of Mesenteric Ischemia/Reperfusion Injury
Source: PLoS One. 2012 Jul 6;7(7):e39934. doi: 10.1371/journal.pone.0039934 (PMC3391230; doi:10.1371/journal.pone.0039934)

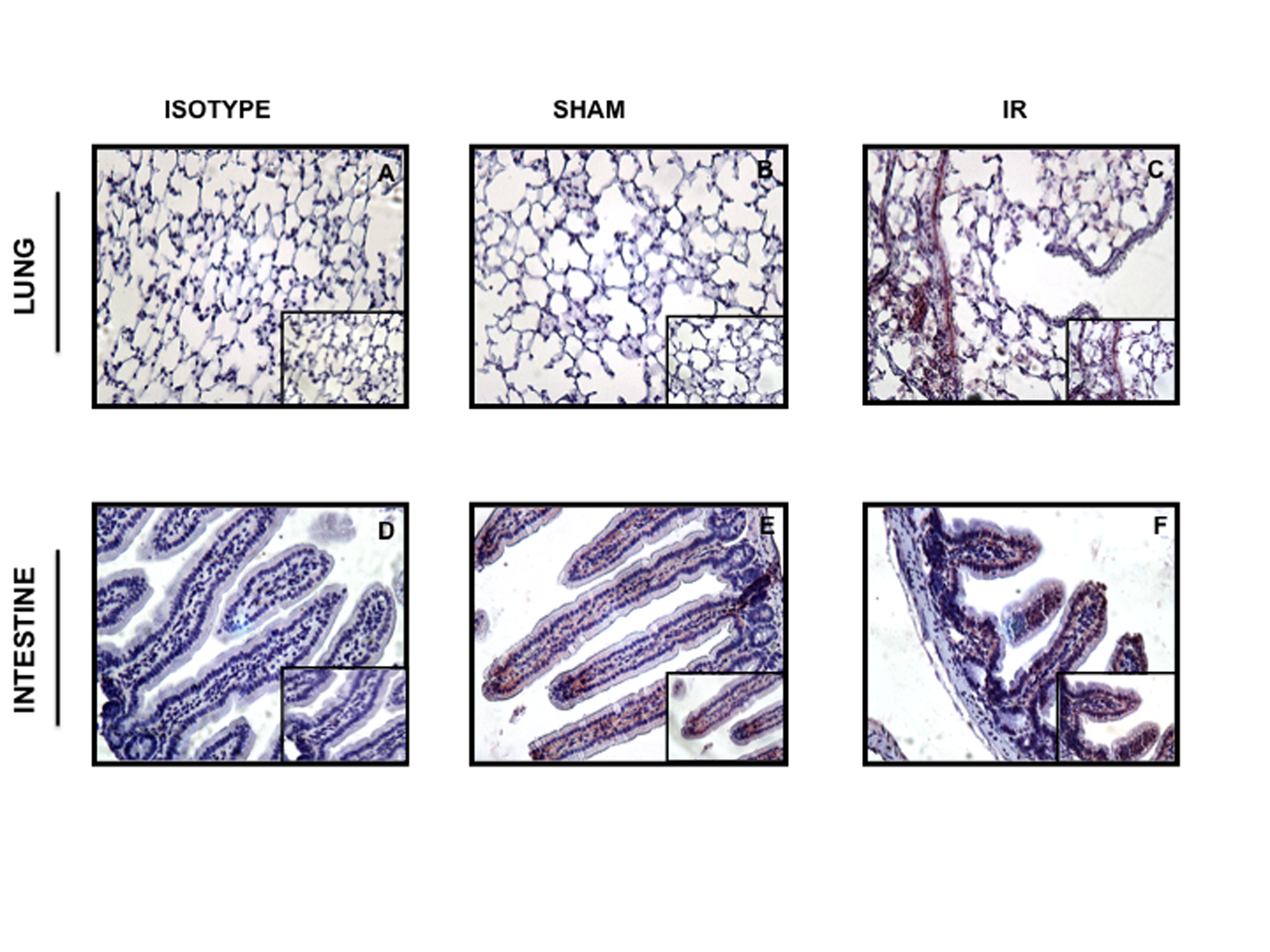

Supplement: Figure S1 — PF4 deposition in the lung and intestine of platelet depleted B6 mice after mesenteric IR injury. Tissue sections of lung (A-C) and intestine (D-F) from platelet-depleted B6 after 30 minutes of mesenteric ischemia and 3 hrs of reperfusion were stained for PF4 (red) and counterstained with hematoxylin (blue). Images are representative of 3–4 mice per group. All images shown are 200× and 400× magnification. (TIF) [file pone.0039934.s001.tif]

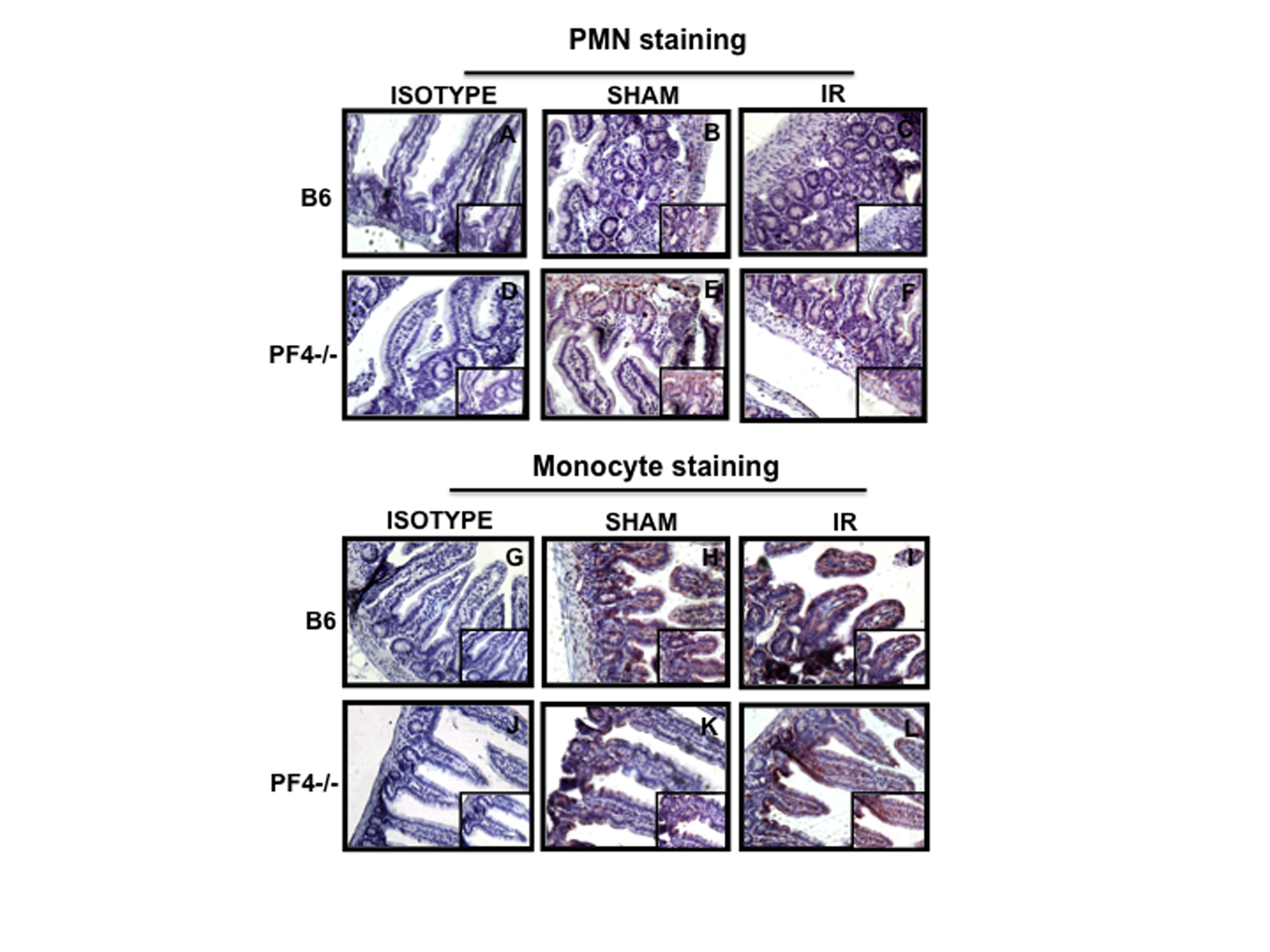

Supplement: Figure S2 — Neutrophil (PMN) and monocyte infiltration in intestine of PF4-/- mice and B6 after mesenteric IR injury. Tissue sections of intestine of B6 and PF4-/- mice after 30 minutes of mesenteric ischemia and 3 hrs of reperfusion and were stained for neutrophils (A-F, red) and monocytes (G-L, red) and counterstained with hematoxylin (blue). A total of 5–8 mice were used for each control and experimental groups in two experiments. All images shown are 200× and 400× magnification. (TIF) [file pone.0039934.s002.tif]
